# Incremental costs of hospital-acquired infections in COVID-19 patients in an adult intensive care unit of a tertiary hospital from a low-resource setting

**DOI:** 10.1186/s13756-023-01240-0

**Published:** 2023-04-21

**Authors:** Aleksa Despotović, Nataša Milić, Anđa Cirković, Branko Milošević, Snežana Jovanović, Vesna Mioljević, Vesna Obradović, Gordana Kovačević, Goran Stevanović

**Affiliations:** 1 Faculty of Medicine, University of Belgrade, Belgrade, Serbia; 2 Department of Medical Statistics and Informatics, Faculty of Medicine, University of Belgrade, Belgrade, Serbia; 3 Division of Nephrology and Hypertension, Mayo Clinic, Rochester, MN USA; 4 Teaching Hospital for Infectious and Tropical Diseases, University Clinical Centre of Serbia, Belgrade, Serbia; 5 Department of Microbiology, University Clinical Centre of Serbia, Belgrade, Serbia; 6 Department of Hospital Epidemiology and Nutrition Hygiene, University Clinical Centre of Serbia, Belgrade, Serbia

**Keywords:** Hospital-acquired infections, Intensive care unit, Medical costs, Economic evaluation, COVID-19, Serbia

## Abstract

**Background:**

Hospital-acquired infections (HAIs) are a global public health problem and put patients at risk of complications, including death. HAIs increase treatment costs, but their financial impact on Serbia’s healthcare system is unknown. Our goal was to assess incremental costs of HAIs in a tertiary care adult intensive care unit (ICU) that managed COVID-19 patients.

**Methods:**

A retrospective study from March 6th to December 31st, 2020 included patients with microbiologically confirmed COVID-19 (positive rapid antigen test or real-time polymerase chain reaction) treated in the ICU of the Teaching Hospital for Infectious and Tropical Diseases, University Clinical Centre of Serbia. Demographic and HAI-specific data acquired in our ICU were collected, including total and stratified medical costs (services, materials, laboratory testing, medicines, occupancy costs). Median total and stratified costs were compared in relation to HAI acquisition. Linear regression modelling was used to assess incremental costs of HAIs, adjusted for age, biological sex, prior hospitalisation, Charlson Comorbidity Index (CCI), and Glasgow Coma Scale (GCS) on admission. Outcome variables were length of stay (LOS) in days and mortality.

**Results:**

During the study period, 299 patients were treated for COVID-19, of which 214 were included. HAIs were diagnosed in 56 (26.2%) patients. *Acinetobacter spp.* was the main pathogen in respiratory (38, 45.8%) and bloodstream infections (35, 42.2%), the two main HAI types. Median total costs were significantly greater in patients with HAIs (€1650.4 vs. €4203.2, *p* < 0.001). Longer LOS (10.0 vs. 18.5 days, *p* < 0.001) and higher ICU mortality (51.3% vs. 89.3%, p < 0.001) were seen if HAIs were acquired. Patients with ≥ 2 HAIs had the highest median total costs compared to those without HAIs or with a single HAI (€1650.4 vs. €3343.4 vs. €7336.9, *p* < 0.001). Incremental costs in patients with 1 and ≥ 2 HAIs were €1837.8 (95% CI 1257.8–2417.7, *p* < 0.001) and €5142.5 (95% CI 4262.3–6022.7, *p* < 0.001), respectively.

**Conclusions:**

This is the first economic evaluation of HAIs in Serbia, showing significant additional costs to our healthcare system. HAIs prolong LOS and influence ICU mortality rates. Larger economic assessments are needed to enhance infection control practices.

## Background

Hospital-acquired infections (HAIs) are a significant public health problem and affect every country and healthcare system worldwide. In the European Union (EU) and the European Economic Area (EEA) alone, 3.8 million patients suffer from HAIs and 8.9 million distinct HAI episodes occur every year in acute care hospitals and long-term health facilities [1, 2]. HAIs are ubiquitously associated with a prolonged length of stay (LOS) and disability-adjusted life-years (DALYs), while putting patients at severe risk for morbidity and in-hospital mortality [2–4]. Although impossible to completely eradicate, a substantial number of HAIs is preventable through proper infection control practices and continuous surveillance [5]. The coronavirus disease 19 (COVID-19) pandemic, a highly transmittable and pathogenic viral infection caused by severe acute respiratory syndrome coronavirus 2 (SARS-CoV-2), has put pressure on healthcare systems and increased rates of HAIs have been reported in both high- and low-resource countries since its beginning [6–8]. The inability to fully implement infection prevention protocols, coupled with steep increases in antibiotic use throughout the pandemic, have only facilitated further development of antimicrobial resistance (AMR), which is now responsible for almost 5 million deaths each year [9]. Pathogens exhibiting multidrug resistance (MDR) are becoming prevalent in the hospital setting, and the proportion of MDR pathogens only continues to increase [10].

Choosing the most effective method of infection prevention and surveillance is, to a large extent, driven by economic evaluations [11, 12]. The financial impact of HAIs is enormous, annually estimated to be up to £2.2 billion in the UK and between $96 and $147 billion in the USA [13, 14]. Despite the economic incentive to implement rigorous infection prevention policies, their primary goal is to prevent as many HAIs as possible and reduce the problem of AMR [15].

HAIs are primarily encountered in intensive care units (ICUs), where patients with multiple comorbidities and indwelling invasive devices are at a higher risk of prolonged LOS and adverse outcomes [16–18]. HAIs are also more frequently caused by MDR pathogens in ICUs with fewer treatment options [19, 20], pointing to antimicrobial stewardship as a critical component of infection control [21, 22].

Serbia is a low-resource country in which the vast majority of the population uses the public health system that fully reimburses costs of hospital treatment across all levels of care. Within this system, HAIs have been investigated through several point-prevalence surveys (PPS) and isolated reports aimed at specific patient populations or pathogens [23–27], but no study to date has investigated the economic aspects of HAIs in ICUs. Our primary goal, therefore, was to investigate the incremental costs brought on by HAIs in patients admitted to the ICU with a diagnosis of COVID-19 during the first year of the pandemic. Secondary goals were to identify HAI types and causative pathogens as part of regular surveillance at our ICU, and provide additional context to the economic evaluation.

## Methods

### Study design

**Fig. 1:**
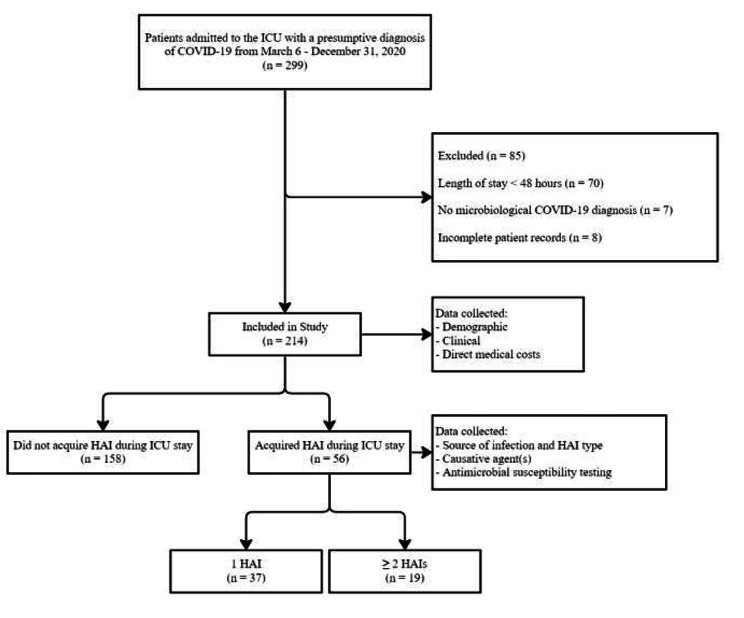
CONSORT Flow Diagram

This retrospective cohort study was conducted at the 16-bed adult ICU of the Teaching Hospital for Infectious and Tropical Diseases, University Clinical Centre of Serbia. All patients with a confirmed microbiological diagnosis of COVID-19 between March 6th and December 31st, 2020 were included in the study. COVID-19 was confirmed either through a positive rapid antigen test and/or viral nucleic acid detection using real-time polymerase chain reaction (RT-PCR) of the upper respiratory tract, through a nasopharyngeal swab test. Exclusion criteria were LOS < 48 hours (n=70), clinical but no microbiological confirmation of COVID-19 (n=7), and incomplete patient records (n=8). As a result, 214 patients were included in the final analysis, as shown by the CONSORT flow diagram (Figure1).

### Hospital-acquired infection definitions

Suspected HAIs were evaluated according to the European Centre for Disease Prevention and Control (eCDC) criteria - occurring ≥ 48 h after admission and the onset of symptoms from day 3, with day 1 being the date of hospitalisation [28]. HAI types that were identified among patients in our study were bloodstream infections (BSI – laboratory confirmed infection with at least one positive blood culture or a combination of clinical symptoms and two positive blood cultures for a common skin contaminant, from two separate blood samples), pneumonia (PN, defined by clinical, radiological, and microbiological criteria across several subcategories), urinary tract infections (UTI), skin and soft tissue infections (SSI), and gastrointestinal infections (GI). BSIs were further analyzed in the presence of a central venous catheter, and classified as central-line-associated BSI (CLABSI) if it was used (even intermittently) in the 48 h preceding the onset of the infection. Similarly, PNs were classified as ventilator-associated pneumonia (VAP) if an invasive respiratory device was present (even intermittently) in the 48 h preceding the onset of the infection [28, 29]. All HAIs were subsequently evaluated using type-specific eCDC criteria [28]. In addition to the first HAI episode, additional HAIs occurring at different sites, either concurrently or subsequently, were reported as separate episodes. Multiple HAIs of the same site were also reported as separate episodes unless the same pathogen was isolated. Pathogen identification and antimicrobial susceptibility testing (AST) was done using Vitek2 ®bioMerieux, based on European Committee on Antimicrobial susceptibility testing (EUCAST) breakpoints and recommendations [30]. Pathogens that were defined as contaminants during microbiology testing by the laboratory were excluded from the analysis. The Magiorakos et al. criteria for multidrug resistance (MDR) assessment was used [31].

### Data Collection

Patient data was collected from the electronic medical record system (EMR) and the following variables were extracted: age, biological sex, prior hospitalisation and LOS (treatment in another another acute care facility before admission to our ICU), date of admission and discharge from our ICU from which LOS was calculated, presence of comorbidities that allowed calculation of the Charlson comorbidity index (CCI) and Glasgow Coma Scale (GCS) score at admission. Data regarding use of invasive devices (urinary/central venous catheter, mechanical ventilation > 48 h, nasogastric tube) and previous antibiotic use (48 h before and after admission) was also collected. Outcome measures were death and LOS in days. If patients developed one or more HAIs at our ICU, data on the tissue from which HAIs were identified (urine, blood, cerebrospinal fluid, endotracheal aspirate, or from the indwelling device – urinary/central venous catheter, or endotracheal tube), as well as the causative agent and AST (when available).

### Economic assessment

Treatment costs were also extracted from the EMR system. Since the our institution is a public hospital, the entire cost of hospitalisation is reimbursed by the Republic Fund of Health Insurance. Information related to direct medical costs were available, including total medical costs, further stratified into following groups: materials; services (nursing/imaging/other procedures such as drug administration, transfusions, obtaining tissue samples for diagnostic purposes, invasive device placement and maintenance, as well as therapeutic procedures such as decubitus ulcer prevention; costs of transfer from another facility, on the other hand, were not available and thus excluded from this group); medications (further divided into antibiotics and non-antibiotic drugs); laboratory testing; and costs related to hospital occupancy (bed and meal). In addition to differences in total and stratified costs, incremental costs were compared - the extent to which HAIs (both 1 and ≥ 2 episodes) added to the overall costs.

Treatment costs were available in Serbia’s local currency, the Serbian Dinar (RSD). To enable their interpretation in EUR, a conversion from RSD to EUR was done using the average annual exchange rate from the National Bank of Serbia (NBS) for the year 2020, which was 117.577 RSD for 1 EUR [32].

### Statistical analysis

For all patients, mean and standard deviation were used to describe normally distributed data, whereas median and minimum-maximum values were used to describe data that did not exhibit normal distribution. Numbers and percentages were used for categorical variables. Depending on the type of variable and its distribution, Chi-square test, Mann-Whitney U test or the independent T-test were used to compare variables among patients with and without HAIs. The Mann-Whitney U test was also used for comparison of median total costs and individual cost groups in patients with and without HAIs, as well as LOS. In addition to comparing median costs, univariate and multivariate linear regression with a confidence interval of 95% was performed to assess the incremental costs due to HAIs, adjusted for key factors - age, biological sex, CCI, prior hospitalisation, and GCS score on admission. Both analyses were done in patients who developed only 1 HAI and in patients who developed ≥ 2 HAIs. For all analyses, statistical significance was established at p < 0.05.

The Statistical Package for Social Sciences (SPSS) software version 23 (IBM Corp. Released 2015. IBM SPSS Statistics for Windows, Version 23.0. Armonk, NY, USA: IBM Corp.) was used for analysis of patient data. Anonymisation of data was ensured prior to the analysis and the study was approved by the Ethics Committee of the University Clinical Centre of Serbia (IRB Number 847/2/2022).

## Results

A total of 214 patients were admitted to our ICU with a microbiologically confirmed diagnosis of COVID-19. Patients were predominantly male (158, 73.8%) and mean age was 63.2 ± 14.7 years. Most frequent comorbidities were diabetes mellitus (72, 33.1%) and congestive heart failure (26, 11.6%). Of 214 patients, 56 (26.2%) developed at least one HAI during their stay at the ICU. Differences in patient characteristics in relation to HAI acquisition are shown in Table 1. Prior hospitalisation in another care facility before admission to our ICU (55.7% vs. 80.4%, p = 0.001) was more frequent among patients who developed HAIs, as was a longer LOS at the previous treatment facility (2.0 vs. 4.0 days, p = 0.002). No differences were observed in GCS score at admission or CCI.

**Table 1:** Characteristics ofpatients admitted to the ICU with COVID-19 in 2020

Variable	All patients (n = 214)	no HAI (n = 158)	HAI (n = 56)	*p*
Age, years	63.2 ± 14.7	63.3 ± 15.3	62.8 ± 13.3	0.836
Biological sex, male	158 (73.8)	113 (71.5)	45 (80.4)	0.196
GCS on admission	12.1 ± 2.8	12.4 ± 2.6	11.5 ± 3.3	0.056
Transferred from another care facility	133 (62.1)	88 (55.7)	45 (80.4)	**0.001**
LOS prior to ICU admission (days)	2.0 (0–30)	2.0 (0–25)	4.0 (0–30)	**0.002**
**Charlson Comorbidity Index**	3.37 ± 2.24	3.41 ± 2.33	3.27 ± 2.02	0.682
Myocardial Infarction	20 (8.9)	15 (9.5)	5 (8.9)	0.901
Congestive Heart Failure	26 (11.6)	23 (14.6)	3 (5.4)	0.070
Peripheral Vascular Disease	3 (1.4)	3 (1.9)	0 (0.0)	0.299
CVI or TIA	11 (4.9)	8 (5.1)	3 (5.4)	0.932
Dementia	10 (4.5)	9 (5.7)	1 (1.8)	0.234
COPD	23 (10.3)	16 (10.1)	7 (12.5)	0.622
Connective Tissue disease	11 (4.9)	9 (5.7)	2 (3.6)	0.536
Peptic Ulcer Disease	5 (2.2)	5 (3.2)	0 (0.0)	0.178
Liver Disease	5 (2.2)	5 (3.2)	0 (0.0)	0.178
Diabetes Mellitus	72 (33.1)	53 (33.5)	19 (33.9)	0.958
With end-organ damage	3 (4.2)			
Hemiplegia	7 (3.1)	6 (3.8)	1 (1.8)	0.467
Moderate/Severe CKD	6 (2.7)	2 (1.3)	4 (7.1)	0.022
Solid Tumor	13 (5.8)	9 (5.7)	4 (7.1)	0.697
Leukemia	4 (1.8)	3 (1.9)	1 (1.8)	0.957
Lymphoma	4 (1.8)	2 (1.3)	2 (3.6)	0.274
HIV infection	7 (3.1)	6 (3.8)	1 (1.8)	0.467
Invasive Device use				
Urinary Catheter	124 (57.1)	70 (44.3)	54 (96.4)	<** 0.001**
Central Venous Catheter	61 (28.5)	20 (12.7)	41 (73.2)	<** 0.001**
Mechanical Ventilation > 48 h	124 (57.1)	68 (43.0)	56 (100.0)	<** 0.001**
Nasogastric Tube	29 (13.5)	11 (7.0)	18 (32.1)	< **0.001**
Previous antibiotic use	164 (77.0)	109 (68.9)	55 (98.2)	**0.009**

HAI - hospital-acquired infection; GCS - Glasgow Coma Scale LOS - length of stay; CCI - Charlson Comorbidity Index; CVA - cerebrovascular insult; TIA - transient ischaemic attack; COPD - chronic obstructive pulmonary disease; CKD - chronic kidney disease; HIV - human immunodeficiency virus;

The use of all invasive devices were associated with HAI development – urinary catheters (44.3% vs. 96.4%, *p* < 0.001); central venous catheter (CVC – 12.7% vs. 73.2%, *p* < 0.001); mechanical ventilation > 48 h (43.0% vs. 100.0%, *p* < 0.001); and nasogastric tube (7.0% vs. 32.1%, *p* < 0.001). Similarly, previous antibiotic was also associated with HAI development (68.9% vs. 98.2%, *p* = 0.009).

Of the 56 patients who developed at least 1 HAI, a third of patients (19, 33.9%) had more than one HAI during their hospitalisation. A total of 83 distinct HAI episodes were identified − 16 patients (28.6%) had ≥ 2 HAIs. Pneumonia (38, 45.8%) was the most common type of HAI, of which the vast majority were VAP (36, 94.7%) (Table 2). Bloodstream infections (35, 42.2%) was the second major group of HAIs, with two thirds classified as CLABSI (23, 65.7%). Gastrointestinal infections, all caused by *Clostridium difficile* (6, 7,2%), urinary tract infections (2, 2.4%) and skin and soft tissue infections (2, 2.4%) were identified as well. No significant differences were observed when looking at the types of HAIs identified in patients who developed 1 or ≥ 2 HAIs.

**Table 2:** Hospital-acquired infection types identified during the study period

HAI type	Total (n, %)	1 HAI episode (n = 37)	≥ 2 HAIs (n = 19)
Pneumonia	38 (45.8)	18 (41.9)	20 (43.4)
*VAP*	36 (94.7)	17 (94.4)	19 (95.0)
BSI	35 (42.2)	18 (41.9)	17 (40.0)
*CLABSI*	23 (65.7)	11 (61.1)	12 (70.6)
GI-CDI	6 (7.2)	0 (0.0)	6 (13.0)
CAUTI	2 (2.4)	1 (2.3)	1 (2.1)
SSI	2 (2.4)	0 (0.0)	2 (4.3)
Total	83 (100)	37 (51.8)	46 (48.2)

HAI – hospital-acquired infection; VAP – ventilation-associated pneumonia; BSI – bloodstream infection; CLABSI – central-line-associated bloodstream infection; GI-CDI – gastrointestinal infection caused by *Clostridium difficile*; CAUTI – catheter-associated urinary tract infection; SSI – skin and soft tissue infection;

Causative agents of HAIs stratified across different types are shown in Table 3. Over a third (29, 38.2%) of HAIs were polymicrobial, and a total of 121 pathogens across 13 species were identified. *Acinetobacter spp.* was most frequently isolated in PNs and in BSIs, whereas coagulase-negative *Staphylococcus* (CoNS) were as frequently identified in BSIs. A substantial number of pathogens were classified as MDR (92, 76.0%).

**Table 3:** Causative agents of HAIs, stratified across HAI types

Isolates	Total	PN	BSI	GI	UTI	SSI
	(n, %)					
*Acinetobacter spp.*	52 (43.0)	36 (65.5)	15 (28.3)		1 (50.0)	
Coagulase-negative *Staphylococcus*	17 (14.0)	2 (3.6)	15 (28.3)			
*Enterococcus spp.*	10 (8.3)	1 (1.8)	6 (11.3)		1 (50.0)	2 (40.0)
*Pseudomonas aeruginosa*	10 (8.3)	7 (12.7)	2 (3.8)			1 (20.0)
*Clostridium difficile*	6 (5.0)			6 (100.0)		
*Klebsiella spp.*	5 (4.1)	2 (3.6)	3 (5.7)			
*Proteus mirabilis*	5 (4.1)	2 (3.6)	1 (1.9)			2 (40.0)
Other Staphylococcal species	3 (2.5)		3 (5.7)			
*Staphylococcus aureus*	3 (2.5)	2 (3.6)	1 (1.9)			
*Diphtheroids*	3 (2.5)	1 (1.8)	2 (3.8)			
*Providencia spp.*	3 (2.5)	2 (3.6)	1 (1.9)			
*Stenotrophomonas maltophilla*	2 (1.7)		2 (3.8)			
*Achromobacter xylooxidans*	2 (1.7)		2 (3.8)			
*Total*	121	55	53	6	2	5

PN - Pneumonia; BSI - bloodstream infection; GI - gastrointestinal infection; UTI - urinary tract infection; SSI - skin and soft tissue infection

**Fig. 2:**
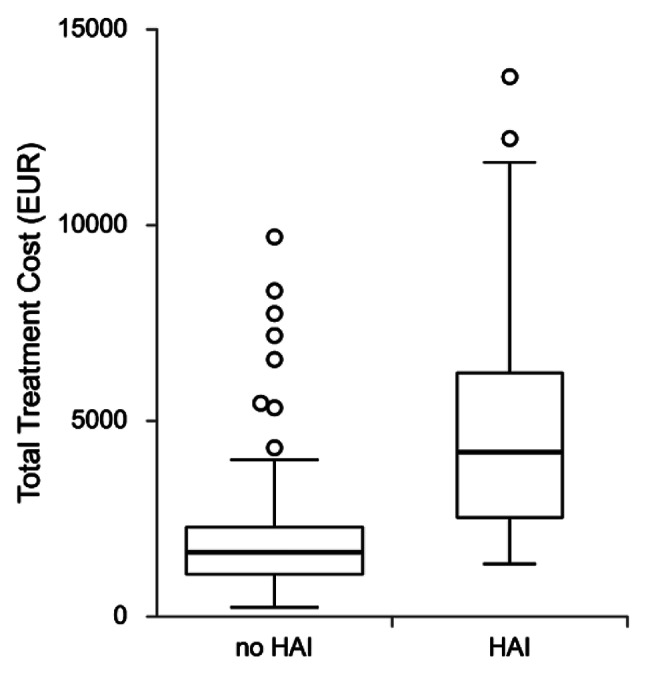
Distribution of total treatment costs in patients with and without HAIs

Median total costs were greater in patients who developed a HAI (€1650.4 vs. €4203.2, *p *< 0.001), and the difference was statistically significant across all subgroups: materials (€106.5 vs. €282.0, *p* < 0.001); services (€423.3 vs. €807.8,* p* < 0.001); laboratory testing (€169.7 vs. €341.5,* p* < 0.001); occupancy costs (€131.4 vs. €249.7,* p* < 0.001); and medications (€728.2 vs. €2284.1, *p* < 0.001), divided further into antibiotics (€130.2 vs. €485.1,* p* < 0.001) and other medications (€568.6 vs. €1633.9,* p* < 0.001). (Table 4). Median length of stay was longer (10.0 vs. 18.5 days, *p* < 0.001), and ICU mortality rates were higher in patients who acquired HAIs (51.3% vs. 89.3%, *p *< 0.001). Figure 2 provides a visual presentation of cost distribution.

**Table 4:** Comparison of costs, LOS and mortality rate in patients with and without HAIs

Variable	no HAI (n = 158)	HAI (n = 56)	*p*
Total costs, €	1650.4 (237–9700.0)	4203.2 (1347–13,792)	**< 0.001**
Materials	106.5 (7.5-478.5)	282.0 (52.7-1073.4)	**< 0.001**
Services	423.3 (82.8-1488.4)	807.8 (326.1-2587.7)	**< 0.001**
Laboratory testing	169.7 (7.8-761.7)	341.5 (144.6-1351.6)	**< 0.001**
Occupancy costs	131.4 (39.4–736.0)	249.7 (78.9-1038.4)	**< 0.001**
Medications	728.2 (50.1-6835.7)	2284.1 (455.9-9205.4)	**< 0.001**
Antibiotics	130.2 (0.0-1089.0)	485.1 (66.0-3047.4)	**< 0.001**
Non-antibiotics	568.6 (30.9-6588.1)	1633.9 (258.9-8406.7)	**< 0.001**
LOS (days)	10.0 (3–55)	18.5 (6–80)	**< 0.001**
ICU Mortality	81 (51.3%)	50 (89.3%)	**< 0.001**

HAI – hospital acquired infection; LOS – length of stay.

**Fig. 3:**
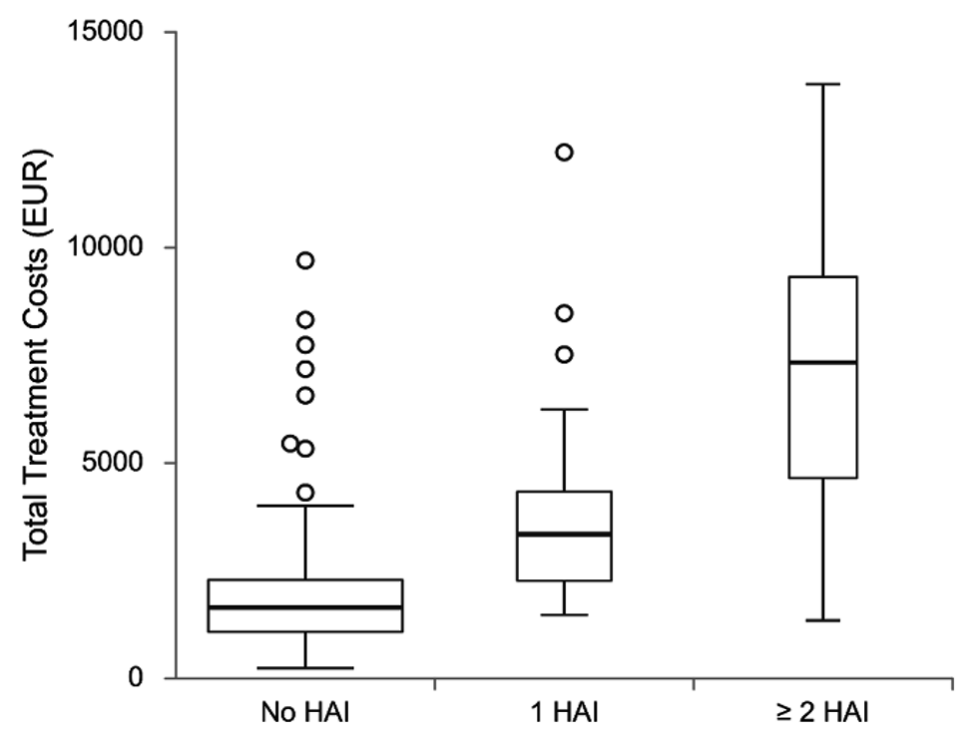
Distribution of total treatment costs in patients with no HAIs, 1 HAI, and ≥ 2 HAIs.

Costs were further analysed based on the number of acquired HAIs, and major differences were seen for both patients who acquired only one HAI and for those who developed ≥ 2 HAIs in the ICU (Table 5). Significant differences in median costs across all subgroups were observed, as well as LOS and mortality. When compared, patients acquiring ≥ 2 HAIs during their hospitalisation had greater total costs and LOS than patients who acquired 1 HAI, but mortality rates were not statistically significant. Distribution of costs across the three groups are shown in Fig. 3 .

**Table 5:** Comparison of hospital costs stratified by number of HAI episodes

Variable	no HAI (n = 158)	1 HAI (n = 37)	*p* ^*a*^	≥ 2 HAIs (n = 19)	*pb*	*p* ^*c*^
Total costs, €	1650.4 (237–9700.0)	3343.4 (1474.0-12211.0)	**< 0.001**	7336.9 (1347.0-13792.0)	**< 0.001**	**< 0.001**
Services	423.3 (82.8-1488.4)	682.7 (326.1-1513.5)	**< 0.001**	1253.0 (503.7-2587.7)	**< 0.001**	**< 0.001**
Materials	106.5 (7.5-478.5)	232.7 (52.7-527.7)	**< 0.001**	342.8 (101.1-1073.4)	**< 0.001**	**0.005**
Laboratory testing	169.7 (7.8-761.7)	305.5 (149.8-896.8)	**< 0.001**	444.8 (144-6-1351.6)	**< 0.001**	**0.027**
Occupancy cost	131.4 (39.4–736.0)	210.3 (78.9-657.2)	**0.004**	407.4 (118.3-1038.4)	**< 0.001**	**0.001**
Medications	728.2 (50.1-6835.7)	1793.3 (493.7-9205.4)	**< 0.001**	4067.7 (455-9-8446.1)	**< 0.001**	**< 0.001**
Antibiotics	130.2 (0.0-1089.0)	358.1 (66.0-1296.3)	**< 0.001**	1226.7 (197.1-3047.4)	**< 0.001**	**< 0.001**
Non-antibiotic drugs	568.6 (30.9-6588.1)	1430.2 (326.4-8406.7)	**< 0.001**	2306.4 (258.9-7437.4)	**< 0.001**	**0.002**
LOS (days)	10.0 (3–55)	16.0 (6–55)	**0.003**	31.0 (9–80)	**< 0.001**	**0.001**
Mortality	81 (51.3%)	35 (94.6%)	**< 0.001**	15 (78.9%)	**< 0.001**	0.079

HAI hospital-acquired infection; LOS length of stay; ^a^ difference between costs in patients without HAIs and with 1 HAI; ^b^ difference between costs in patients with 1 HAI and in ≥ 2 HAIs; ^c^difference between costs in patients with 1 and  ≥ 2 HAIs

Using linear regression adjusted for age, biological sex, CCI, and prior hospitalisation, patients who developed a single episode of HAI (Table 6a) carried incremental costs of €1837.8 (95% CI 1257.8–2417.7, *p* < 0.001), whereas in patients with ≥ 2 HAIs (Table 6b), incremental costs were €5142.5 (95% CI 4262.3–6022.7, *p* < 0.001). Age was also a statistically significant predictor of cost in the latter group (− 27.1, 95% CI -50.7 to -3.5, *p* = 0.025).

**Table 6:** a Multivariate regression analysis of incremental costs for patients with one HAI

Variable	Multivariate analysis		
	B	95% CI	*p*
1 HAI	1837.8	1257.8–2417.7	**< 0.001**
Age	-19.0	-38.4–0.5	0.056
Biological sex (male)	-295.2	-818.2–227.8	0.267
CCI	62.9	-63.5–189.3	0.327
Prior Hospitalization	239.1	-230.0–708.2	0.316
GCS Score on admission	62.2	-33.8–158.2	0.203

HAI - hospital-acquired infection; CCI - Charlson Comorbidity Index; GCS - Glasgow Coma Scale; B – regression coefficient that corresponds to cost increase or decrease in € ; CI - confidence interval;

**Table 6:** b Multivariate regression analysis of incremental costs for patients with ≥ 2 HAIs

Variable	Multivariate analysis		
	B	95% CI	*p*
≥ 2 HAIs	5142.5	4262.3–6022.7	**< 0.001**
Age	-27.1	− 50.7 to − 3.5	**0.025**
Biological sex (male)	-285.8	-893.9–322.4	0.355
CCI	84.0	-67.2–235.3	0.274
Prior Hospitalization	112.8	-450.3–675.8	0.693
GCS Score on admission	-14.7	-126.2–96.8	0.794

HAI - hospital-acquired infection; CCI - Charlson Comorbidity Index; GCS - Glasgow Coma Scale; B – regression coefficient that corresponds to cost increase or decrease in €; CI - confidence interval;

## Discussion

The rate of HAI occurrence in our ICU (26.2%) is higher than pre-pandemic data from European countries, where 19.2% of patients had at least 1 HAI [1]. Although the rate is higher, these findings are not surprising since a clear spike in HAIs has been globally documented during the first and second wave of the COVID-19 pandemic, both included in our study period [6, 33, 34]. Strict implementation of infection control practices, however, can effectively reduce HAI occurrence and its impact even in subsequent waves [35], proving the utility of measures aimed at healthcare staff practices and habits.

Pneumonia was the most prevalent HAI type before the pandemic [36], and the frequent need for intubation and mechanical ventilation in COVID-19 patients only facilitated VAP development in our ICU [37]. Also, the increased presence of BSIs among COVID-19 patients, of which CLABSIs comprised a majority of infections, is supported by reports from other countries dealing with HAIs during the pandemic [38]. Despite the well-established risks of invasive device use and HAI development, particularly VAP and CLABSI, such high numbers mandate a revision of procedures related to their placement, maintenance, and overall hygiene procedures in our ICU, coupled with additional education efforts.

More importantly, we observed a significant difference in the profile of causative agents compared to most European countries, both prior and during the pandemic. *Acinetobacter spp.* comprised 65.5% of pathogens identified in PNs and these findings are only comparable to Romania (39.5%), with all other countries isolating this pathogens in ≤ 20% of cases [36]. Though *Acinetobacter spp.* is increasingly recognized as one of the main causative agents of HAIs in Central Europe and Eastern Asia [39], such high numbers warrant further exploration. Because of its ubiquitous presence, ability to survive in the hospital environment, and a very high resistance rate to most antibiotics [40], healthcare staff education pathogen-specific measures, but also increasing staff capacity to support the increased demand should be the direction of interventions toward reducing *Acinetobacter spp.* presence and HAIs in general. Conversely, *Klebsiella spp.* (5.8%) was less prevalent, with rates as high as 36.7% in Slovakia and 27.3% in Estonia [36]. The presence of *Pseudomonas aeruginosa* (18.4%) was in line with the findings from other countries. Similar to PNs, rate of *Acinetobacter spp.* isolation in BSIs is only comparable to Romania (19.3%) [36]. Identifying CoNS as one of the main causes of BSIs is in agreement with studies from most countries, including Belgium, Spain, Germany, and Lithuania [36].

The causative agents of HAIs in our ICU are also different when looking at HAI data published during the pandemic. Reports from Italy show similar pathogen profiles in COVID-19 patients seen prior to the pandemic, with *Acinetobacter spp.* isolated in < 8% for both PNs and BSIs [41]. Similar results were found in Spain and Brazil [42, 43], and point to a clear need for improvement in infection control practices aimed at reducing *Acinetobacter spp.* presence in our ICU. In our setting, the COVID-19 pandemic brought additional pressure on underresourced staff, affecting the quality of infection control measures and subsequently leading to more frequent occurrences of pathogens that have already been present in our ICU. As mentioned previously, the focus of intervention in our ICU should be aimed at better healthcare staffing and appropriate management, given its clear benefit on HAI reduction [44].

Most EU countries have reduced the overall rate of antibiotic use in the last decade [45], but Serbia continues to struggle with the disproportionate rate of antimicrobial consumption driven by ease of access, lack of education, and the ability to self-medicate [46–48]. In fact, the latest results from our country show a steady increase in antibiotic use prior to the pandemic [49], whereas very high rates of antibiotic prescribing during the pandemic continues to cast doubt on the current guidelines and recommended practices [50]. These findings, in turn, are not surprising when we found 76% of identified pathogens to be MDR. With the exception of one study in the US where 100% of isolates from HAIs in COVID-19 patients were MDR [51], studies from Belgium, Qatar, Italy, Pakistan, and China show the rate of MDR pathogens between 2.8% and 56% [52], all significantly lower than our numbers. Antimicrobial use has been particularly troubling during the COVID-19 pandemic [53, 54], and will only facilitate antimicrobial resistance, both in the community and in the hospital setting. As our study results point to very high rates of MDR pathogens, approval of new antibiotics for complicated HAIs are a critical infection control measure. These drugs have been on the EU market for years and have proven to be sucessful in treating various forms of HAIs [55, 56]. The local health authorities must facilitate their introduction into standard medical practice to prevent further growth of AMR and the burden of HAI-related complications on patients and the public healthcare system.

The absence of economic evaluations related to HAIs and antimicrobial resistance could be one of the reasons why this public health issue is hard to address in our country. A recent study looked at medical costs of treating COVID-19 patients [57], but without looking into potential differencess with respect to HAI occurrence. The data presented here are, in fact, the first to look at costs associated with HAIs in ICUs in Serbia. These findings clearly show what many other countries have already established – HAIs greatly increase costs of overall treatment, especially if multiple episodes occur [13, 58–61]. HAIs in our ICU carry a several-fold increase in median total costs, distributed across all cost categories. The biggest relative difference was observed for antibiotic spending, where a 10-fold increase in median cost occurred if patients acquired ≥ 2 HAIs (€130.2 vs. €1226.7, p < 0.001).

Similarly to the NHS system in the UK, the burden on HAIs in Serbia falls on the public healthcare system and the national budget, as it fuly reimburses 100% of treatment costs, including complications that developed as a result of HAIs. Risk mitigation through financial means such as “never-events” in the US where expenses related to HAIs are not reimbursed [62], are unlikely to be effective in a public healthcare system. Instead, the focus should be on uncovering the full extent to which additional costs of HAIs affect the public health domain and design interventions to reduce that burden. The UK estimates that 99.8% of HAI-related costs are related to patient management and cite the increased awareness of the impact HAIs have on patients and the system as key drivers of clinical and economic benefit [63]. Our results show incremental costs in patients developing 1 or ≥ 2 HAIs to be €1837.8 and €5142.5, respectively, marking the first step in understanding costs associated with HAIs in our country. The drivers of cost, based on the study results, can be attributed to more frequent use of invasive devices and accompanying procedures, as well as materials and services related to their maintenance, but also medications to treat HAIs and longer LOS. Our findings should incentivise other ICUs at the University Clinical Centre of Serbia that collectively treat thousands of patients every year to perform similar analyses, given that the EMRs now allow such studies. The same can be stated for local authorities, who could start performing economic evaluations as part of standard hospital management. specifically aimed at uncovering the incremental costs of HAIs on a larger scale. Various frameworks for infection control have already been extensively described and used in different settings [12]. Earlier testing and rigorous hygiene practices are examples of interventions [64, 65], but virtually all of the strategies described in literature, with proper planning and implementation based on economic evaluations, improve survival rates and reduce costs. Serbia’s “Guide for Prevention, Early Detection and Control of Hospital-Acquired infections” defines nation-wide practices for HAI prevention, surveillance, and operations. Despite their place in HAI management, economic evaluations are, unfortunately, not mentioned in this guide. More work needs to be done to incorporate the economic aspect of HAIs in our policies and guidelines, mainly through education of the heatlhcare management staff and government officials. Though our study did not include the calculation of DALYs and the indirect impact of HAIs, their use in estimating the burden of HAIs has been widespread, and could be considered a viable method of assessment.

In addition to the pure economic impact, the median LOS was greatly prolonged in patients who acquired HAIs. In fact, the median LOS was three times higher in patients who acquired ≥ 2 HAIs (10.0 vs. 31.0, p < 0.001), and is driven by HAI occurrence to a large extent [13]. Increased resource utilisation is known to contribute to HAI-related mortality [66], but further insight is needed into the analysis of resource utilisation in our facility. In any case, prolonged hospitalisation caused by HAIs is detrimental during times of scare hospital capacity and resources, as was the case with COVID-19 where HAIs were reported at a higher rate [43, 67]. Lastly, ICU mortality rate was substantially higher in patients who acquired HAIs. As studies continue to emphasise the risk HAIs pose for patients [3], our country needs to uncover the true burden of HAIs and associated morbidity and mortality as a consequence of their acquisition.

Our study had several limitations. First, the retrospective study design limited our capacity to analyse HAIs in greater detail, primarily in determining costs attributed to each HAI type. As our EMR system does not allow retrospective analysis of costs on a daily basis, calculating costs related to specific types of HAIs (such as VAP, or CLABSI), was not feasible. Furthermore, the study design also limited us in including direct medical costs only, as indirect costs such as working hours lost due to HAIs or DALYs, were not within the study scope, even though their absence underestimated the true cost of HAIs. As economic parameters calculated in our study are harder to compare with the majority of European countries, incorporating DALYs and relevant metrics in subsequent study designs would allow a direct comparison. Third, the overall sample size was relatively small and collected from only one ICU, which makes it hard to establish more generalised conclusions. Studies like these should be conducted in other ICU types (surgical, neonatal) and in those managing different types of patients and diseases, so that a more accurate financial assessment of HAIs within our healthcare system can be made.

## Conclusions

Our study showed that COVID-19 patients during the first and second waves of the pandemic who acquire HAIs in the ICU have considerably higher costs of treatment, stay longer in the hospital, and have a higher ICU mortality rate. To reduce the financial burden of HAIs, large-scale economic evaluations need to be conducted and serve as the basis for designing better infection control practices, primarily education of hospital staff and better resource utilisation policies. Antimicrobial stewardship and surveillance efforts must be rigorously implemented to reduce the problem of antimicrobial resistance. HAIs require a multidisciplinary approach in order to reduce their effects on patients, including stakeholders from the public health institutions and the government, especially during a global health emergency such as the COVID-19 pandemic.

## Data Availability

The datasets generated and analysed during the study are available from the corresponding author (Aleksa Despotović: alexadespotovic21@gmail.com) on reasonable request.

## Abbreviations

ICU: Intensive care unit
HAI: Hospital-acquired infection
PCR: Polymerase chain reaction
GCS: Glasgow Comma Scale
CCI: Charlson Comorbidity Index
LOS: Length of stay
CVA: Cerebrovascular insult
TIA: Transient ischaemic attack
COPD: Chronic obstructive pulmonary disease
CKD: Chronic kidney disease
HIV: Human immunodeficiency virus
PN: Pneumonia
VAP: Ventilation-associated pneumonia
BSI: Bloodstream infection
CLABSI: Central line-associated bloodstream infection
GI: Gastrointestinal infection
UTI: Urinary tract infection
SSI: Skin and soft tissue infection
CoNS: Coagulase-negative *Staphylococcus*. CDI:*Clostridium difficile* infection
